# Assessing the relationship between syringe exchange, pharmacy, and street sources of accessing syringes and injection drug use behavior in a pooled nationally representative sample of people who inject drugs in the United States from 2002 to 2019

**DOI:** 10.1186/s12954-021-00565-6

**Published:** 2021-11-17

**Authors:** Phillip L. Marotta, Kristi Stringer, Leo Beletsky, Brooke S. West, Dawn Goddard-Eckrich, Louisa Gilbert, Tim Hunt, Elwin Wu, Nabila El-Bassel

**Affiliations:** 1 Brown School, Washington University in St. Louis, St. Louis, MO 63130 USA; 2 School of Social Work, Columbia University, New York, NY 10027 USA; 3 Northeastern University, Boston, MA 02115 USA

**Keywords:** People who inject drugs, Syringe access, Injection drug behavior, Syringe exchange programs, Pharmacy

## Abstract

Provision of sterile syringes is an evidence-based strategy of reducing syringe sharing and reusing and yet, access to sterile syringes through pharmacies and syringe exchange programs (SEPs) in the United States remains inadequate. This nationally representative study examined associations between obtaining syringes from pharmacies, SEPs, and sterilizing syringes with bleach and risk of syringe borrowing, lending and reusing syringes in a pooled cross-sectional dataset of 1737 PWID from the 2002–2019 National Survey on Drug Use and Health. Logistic regression was used to produce odds ratios (OR) of the odds of injection drug behaviors after adjusting for obtaining syringes from SEPs, pharmacies, the street, and other sources and potential confounders of race, ethnicity, sex, education, and insurance coverage. Obtaining syringes through SEPs was associated with lower odds of borrowing (OR = .4, CI_95%_ = .2, .9, *p* = .022) and reusing syringes (OR = .3, CI_95%_ = .2, .6, < .001) compared to obtaining syringes on the street. Obtaining syringes from pharmacies was associated with lower odds of borrowing (OR = .5, CI_95%_ = .3, .9, *p* = .037) and lending (OR = .5 CI_95%_ = .3, .9, *p* = .020) syringes. Using bleach to clean syringes was associated with increased odds of borrowing (OR = 2.0, CI_95%_ = 1.3, 3.0, *p* = .002), lending (OR = 2.0, CI_95%_ = 1.3, 3.0, *p* = .002) and reusing syringes (OR = 2.4, CI_95%_ = 1.6, 3.6, *p* < .001). Our findings support provision of syringes through pharmacies and SEPs as a gold-standard strategy of reducing sharing and reuse of syringes in the US.

## Introduction

The United States (US) is home to more than 6.5 million people who inject drugs (PWID) of which an estimated 1 million recently injected drugs [21]. Prior literature suggests between 35 and 47% of people who inject drugs (PWID) share syringes and 58–69% share injection equipment [1, 25]. Over the past 15 years, the precipitous growth in injection of opioids dramatically heightened risk of acquiring human immunodeficiency virus (HIV) and hepatitis C (HCV) [19, 26, 32]. From 2004 to 2014, incidence of HCV increased by 400% (0.4–2.0) among people aged 18–29 and 325% among people aged 30–39 [2]. As the US faces its worst epidemic of opioid use in its history and increasing rates of injecting other types of drugs, barriers persist to accessing sterile syringes that reduce exposures to blood borne pathogens that transmit HIV/HCV and other infectious diseases [19, 26, 32]

Prior literature suggests that PWID who experience difficulty accessing sterile syringes are more likely to engage in borrowing (receptive syringe sharing) lending and reusing syringes [10, 20, 33]. PWID who obtain syringes on the street, through drug distributers and friends increase risk of obtaining previously used syringes and borrowing and lending used syringes to other PWID within drug-using social networks. Reusing syringes introduces the potential for exposures to blood borne pathogens and thus the Centers for Disease Control (CDC) recommends use of new sterile syringes at every injection [2]. Harm reduction practices involving syringe distribution mitigate negative consequences of injection drug use by reducing syringe sharing and reusing syringes through increasing access to sterile syringes and syringe disposal among PWID [10].

Syringe exchange programs (SEP) and pharmacy sales are two predominant syringe access strategies to reduce sharing and reuse of syringes among PWID. SEPs distribute sterile syringes and collect used syringes to prevent borrowing, lending and reusing syringes among PWID [10]. Prior literature suggests that accessing syringes through SEPs greatly reduces syringe sharing behaviors among PWID [10, 16]. Pharmacy distribution involves Over-The-Counter (OTC) purchase of sterile syringes, and the collection of used syringes at pharmacies to attenuate syringe sharing among PWID Several prior studies suggest that providing syringes at pharmacies is associated with lower risk of HIV infection, syringe sharing and reusing syringes [13, 15, 27, 31, 39]. In the absence of access to sterile syringes at SEPs and pharmacies, PWID may disinfect syringes with bleach prior to lending, borrowing or reusing syringes [30]. Although the CDC recommends that all PWID use new and sterile syringes for each injection, sanitation through pure bleach is endorsed as an effective strategy of reducing exposures to blood-borne pathogens particularly when sharing and reusing syringes [2]. Therefore, people who use bleach to sterilize syringes may reuse the same syringe either for themselves or lend the syringe to others compared to PWID who have access to sterile and safe SEPs [30].

Research is lacking using nationally representative datasets that estimate the association between obtaining syringes from SEPs and pharmacies, cleaning syringes with bleach and lending, borrowing and reusing syringes. The nationally representative nature of the analysis enriches a robust body of research over the decades by addressing limitations in existing literature that includes small sample sizes covering short time periods with samples that are not generalizable to the US population. Moreover, prior literature has disproportionately focused on urban populations from single or few locations in the US i.e. [14, 36, 37]. Research is yet to examine the relationship between sources of syringes and outcomes of syringe sharing, reusing syringes and syringe borrowing using the National Survey on Drug Use and Health (NSDUH) which is the largest nationally representative study of drug use in the United States. Most of the existing body of literature focuses on the impact of SEPs, pharmacies and other sources on syringe sharing yet fewer studies include reusing syringes. Our study expands on prior research by investigating the relationship between obtaining syringes at pharmacies, SEPs, the street and other sources, and behaviors of lending, borrowing, and reusing syringes.

To address gaps in the literature, we examined the association between accessing syringes from pharmacies, SEPs, the street and cleaning syringes with bleach and engaging in lending, borrowing and reusing syringes among PWID in the United States from 2002 to 2019. We hypothesized that obtaining syringes at last injection from an SEP and pharmacy would be associated with decreased risk of borrowing, lending and reusing syringes after adjusting for potential confounders. We hypothesized that sterilizing syringes at last injection with bleach would be associated with syringe sharing and reusing syringes.

## Methods

### Data and procedures

Data for this study come from the 2002–2019 annual surveys of the National Survey of Drug Use and Health (NSDUH) amounting to 1,005,421 participants (SAMHSA, 2020). Restricting the sample to participants who reported injecting in the past year and excluding missing data produced a final sample of 1737 PWID.

### Dependent variables

#### Syringe sharing behaviors

*Syringe borrowing* consisted of a question that asked: *“the last time you used a needle for injecting drugs, did you use a needle that you knew or suspected someone else had used before?” Syringe lending* consisted of a question that asked: *“the last time you used a needle for injecting drugs, did someone else use the needle after you?” Reusing syringes* included a question that asked: *The last time you used a needle for injecting drugs, did you use bleach to clean the needle before you used it? Cleaning with bleach* included a question that asked: *“the last time you used a needle for injecting drugs, did you use bleach to clean the needle before you used it?”* Each of these questions are not entirely mutually exclusive and were thus created as separate dichotomous variables.

#### Independent variables

*Sources of syringes* consisted of a question that asked participants where they obtained syringes at last injection. A categorical variable was created with 4 classes of: (1) pharmacy, (2) SEP, (3) other sources and (4) on the street. The category indicating other sources contained small numbers of responses such as a shooting gallery, friend/acquaintance, relative, drug distributor, found in waste can, stolen from medical facility, work or unspecified place, a party, online or internet, given/stolen from a friend, acquaintance, drug dealer, medical facility farm or veterinarian facility and purchased online.

*Covariates* included having health insurance coverage, (Medicaid/medicare, private health insurance), types of drugs injected in the past year (heroin or cocaine), race (Black, White, Asian, Native American, other/more than one race), ethnicity (Hispanic), female sex, and less than high school education. Selection of covariates for this study are based on prior research that suggests PWID who are black, younger of age and impoverished are significantly more likely to engage in syringe sharing behaviors compared to white, wealthier, and older PWID [4, 6, 10, 23].

### Statistical analyses

Descriptive statistics included overall proportions %(n) means (M) with standard errors (SE) and chi-square tests of significant differences between sources of syringes (pharmacy, SEP, the street and other), cleaning with bleach and receptive and distributive syringe sharing and reusing syringes among PWID using Stata Version 17 [34]. All models adjusted for having no health insurance, race, ethnicity, female sex and less than high school education. The parameter coefficients are expressed as odds ratios (OR) with 95% confidence intervals (CI). Survey weights were applied to all bivariate and multivariable analyses and are presented in Table 2. The reference category estimating associations between sources of syringes and injection drug behaviors is obtaining the syringe on the street. Dummy variables were included to adjust for year of data collection in the analysis to adjust for how harm reduction practices changed from 2002 to 2019. None of the year dummy variables were significant in the regression results (results available upon request). All percent frequencies, and parameter estimates were calculated using survey weights provided in the dataset (NSDUH, 2020). Cases with missing variables (< 3%) were excluded. A sensitivity analysis was performed that tested interactions between confounders and behavioral variables (obtaining syringes from SEP, pharmacies, street and other) and no significant findings were observed (results available upon request).

## Results

### Sociodemographic variables

Table 1 presents descriptive sociodemographic and drug use characteristics of the sample. More than three quarters of the sample was White (77.0%, *n* = 1361), followed by Black (7.3% *n* = 79) and Other Race (2.8%, *n* = 105). Hispanic ethnicity accounted for 12.9% (*n* = 181) of the sample. More than a quarter of the sample had less than a high school education (27.0% *n* = 459) and were between the age of 18 and 25 (25.2%, *n* = 930). A majority of the sample were males (68.0%, *n* = 1039). The majority of PWID reported injecting heroin (82.6%, *n* = 1458) followed by almost half who reported injecting cocaine (46.2%, *n* = 789) and more than a third did not have health insurance (33.3%, *n* = 584). Approximately a fifth of the sample of the sample reported lending syringes (19.1%, n = 338) and 16.7% (n = 294) reported borrowing syringes at last injection. More than half (52.7%, *n* = 953) of the sample purchased their last syringe at a pharmacy, 12.49% (*n* = 172) obtained their last syringe at an SEP, 15.6% (*n* = 226) on the street and 19.3% (*n* = 341) through other means. More than a quarter of the sample used bleach to clean their needle at last injection (27.5%, *n* = 415).

**Table 1:** Descriptive characteristics of covariates among PWID from 2002 to 2019 (*n* = 1737)

	%	*n*
Syringe sharing		
Borrowing	16.7	(294)
Lending	19.1	(338)
Reuse syringe	63.1	(1062)
*Source last syringe*		
Got last needle through SEP	12.4	(217)
Purchased last needle at pharmacy	52.7	(953)
Obtained syringe on the street	15.6	(226)
Obtained syringe other means	19.3	(341)
Use bleach to clean last needle	27.5	(415)
*Covariates*		
No insurance coverage	33.3	(584)
*Injection drug use*		
Heroin	82.6	(1458)
Cocaine	46.2	`(789)
18–25 years old	25.2	(930)
*Race*		
Black	7.3	(79)
White	77.0	(1361)
Other race	2.8	(116)
Hispanic	12.9	(181)
Male sex	68.0	(1039)
Less than high school	27.1	(459)

### Sources of obtaining syringes

Table 2 presents bivariate relationships between access to syringes, using bleach to clean last needle, covariates, and engagement in borrowing, lending and reusing syringes. A smaller proportion of participants who obtained syringes from SEPs reported reusing syringes compared to participants who did not obtain syringes from SEPs (10.3%, *n* = 92 vs. 15.8%, *p* = 0.041). The proportion of participants who obtained syringes from a pharmacy was significantly less among participants who engaged in syringe borrowing (43.6%, *n* = 122 vs. 54.8%, *n* = 748, *p* = 0.013), and lending (43.1%, *n* = 159 vs. 55.0% *n* = 794, *p* = 0.012) compared to participants who did not borrow or lend syringes. A greater proportion of participants who borrowed (23.0%, *n* = 60, vs. 14.1%, *n* = 166, *p* = 0.013), lent (23.1%, *n* = 64 vs. 13.8%, *p* = 0.010) and reused syringes (19.2%, *n* = 171 vs. 9.5% *n* = 53, *p* = 0.014) reported obtaining syringes on the street compared to participants who did not share or reuse syringes. A significantly greater proportion of participants who engaged in syringe borrowing (40.1%, *n* = 111 vs. 24.5% *n* = 304, *p* < 0.001), lending (39.8%, *n* = 130 vs. 24.6%, *n* = 262, *p* < 0.001) and reusing syringes (32.8% *n* = 308 vs. 18.8% *n* = 96 *p* = 0.008) reported cleaning their syringes with bleach compared to participants who did not share or reuse syringes.

**Table 2:** Weighted Bivariate tests of differences between sources of syringes, cleaning with bleach and receptive syringe sharing, distributive syringe sharing and reusing syringe at last injection 2002–2019 (*n* = 1737)

	Borrowing			Lending			Reuse syringe		
	Yes *n* (%)	No *n* (%)	*p* value	Yes *n* (%)	No *n* (%)	*p* value	Yes *n* (%)	No *n* (%)	*p* value
Overall									
Access to syringes									
Got last needle through SEP	9.4(27)	13.5(190)	.310	11.1(36)	12.7(181)	.628	10.3(111)	15.8(106)	**.041**
Purchased last needle at pharmacy	43.6(122)	54.8(831)	**.013**	43.1(159)	55.0(794)	**.012**	52.1(569)	53.8(384)	.661
Obtained syringe on the street	23.0(60)	14.1(166)	**.022**	23.1(64)	13.8(162)	**.016**	19.2(171)	9.5(55)	**.001**
Obtained syringe other means	25.0(85)	18.2(256)	.075	22.7(79)	18.5(262)	.250	20.9(211)	18.3(130)	.381
Use bleach to clean last needle	40.1(111)	24.5(304)	**< .001**	39.8(130)	24.6(285)	**< .001**	32.7(308)	18.4(107)	**< .001**
Uninsured	34.1(91)	33.2(493)	.850	38.3(117)	32.1(467)	.167	34.7(367)	31.0(217)	.286
Injection drug use			.990			.239			**< .001**
Heroin	54.0(154)	53.8(794)		47.3(163)	55.4(785)		51.1(540)	58.5(408)	
Cocaine	17.2(44)	17.5(235)		19.2(51)	16.7(228)		14.3(145)	22.7(134)	
Both	28.9(96)	28.8(414)		33.6(124)	27.65(386)		34.7(377)	18.8(133)	
Race									
Black	7.1(11)	7.4(68)	.914	8.3(15)	7.1(64)	.671	6.28(40	9.2(39)	.224
White	73.5(216)	77.7(1145)	.353	73.0(256)	77.9(1105)	.273	76.4(844)	78.1(517)	.613
Other	2.6(22)	2.8(94)	.838	1.2(18)	3.1(98)	.010	2.7(64)	2.8(52)	.862
Hispanic	16.9(45)	12.1(136)	.213	17.5(49)	11.9(132)	.139	14.7(114)	9.9(67)	**.078**
Age 18–25	28.9(172)	24.4(758)	.181	29.5(205)	24.2(725)	.100	24.0(554)	27.2(376)	.210
Less than High School Education	26.7(81)	27.1(378)	.930	32.2(94)	25.73(365)	.132	27.3(292)	26.5(167)	.830
Poverty	43.4(113)	32.6(439)	.022	40.2(119)	33.0(433)	.120	35.9(349)	31.9(206)	.267
Male sex	61.6(139)	69.3(900)	.067	61.3(163)	69.6(876)	**.045**	67.8(623)	68.5(416)	.809

Bold indicates significance at *p* < .05

*p < .05, **p < .01, ***p < .001; All percentages are weighted using the sampling methodology used in the study

### Logistic regression analyses of association between sources of most recent syringe, cleaning syringes with bleach and lending, borrowing and reusing syringes

With a reference category of obtaining syringes on the street, obtaining the most recent syringe used for injection through an SEP was associated with lower adjusted odds (aOR) of syringe borrowing (aOR = 0.4, 95%CI = 0.2, 0.9, *p* = 0.022), and reusing syringes (aOR = 0.3, 95% CI = 0.2, 0.6, *p* < 0.001) after adjusting for potential confounders of injecting cocaine or heroin, race, ethnicity, less than high school education, poverty, age between 18 and 25, and injection of cocaine, heroin or both. Using a pharmacy to purchase most recent syringe used for injection was associated with lower odds of borrowing (aOR = 0.5, 95% CI = 0.3, 0.9, *p* = 0.037) and lending (aOR = 0.5, CI = 0.3, 0.9. *p* = 0.020) syringes. Cleaning syringes with bleach prior to injection was associated with increased odds of borrowing (aOR = 2.0, 95% CI = 1.3, 3.0, *p* = 0.002), lending (aOR = 2.0, 95% CI = 1.3, 3.0, *p* = 0.002) and reusing syringes (aOR = 2.4, 95% CI = 1.6, 3.6, *p* < 0.001) (Table 3).

**Table 3:** Logistic regression results of sources of syringes, using bleach to clean syringes and risk factors for syringe sharing and reusing syringes 2002–2019 (*n* = 1737)

	Borrowing			Lending			Reuse syringe		
	aOR	95% CI	*p* value	aOR	95%CI	*p* value	aOR	95%CI	*p* value
Access to syringes									
SEP	.4	(.2, .9)	**.022**	.5	(.2, 1.1)	.073	.3	(.2, .6)	** < .001**
Pharmacy	.5	(.3, .9)	**.037**	.5	(.3, .9)	**.020**	.5	(.3, .9)	**.016**
Other means	.91	(.5, 1.7)	.773	.8	(.4, 1.4)	.423	.5	(.3, .9)	**.025**
Street	Ref	Ref	Ref	Ref	Ref	Ref	Ref	Ref	Ref
Use bleach to clean last needle	**2.0**	**(1.3, 3.0)**	**.002**	2.0	(1.3, 3.0)	**.002**	2.4	(1.6, 3.6)	**< .001**
No insurance	1.0	(.7, 1.6)	.933	1.3	(.8, 1.9)	.249	1.2	(.9, 1.7)	.274
Race									
Black	.9	(.3, 2.4)	.834	1.1	(.5, 2.4)	.826	.5	(.2, 1.1)	.08
Other race	.9	(.4, 2.1)	.914	.4	(.1, .9)	**.027**	1.1	(.6, 2.0)	.779
White	Ref	Ref	Ref	Ref	Ref	Ref	Ref	Ref	Ref
Hispanic ethnicity	1.4	(.7, 2.6)	.316	1.5	(.8, 2.7)	.184	1.3	(.7, 2.1)	.394
Male sex	.8	(.5, 1.1)	.147	.7	(.5, .9)	**.046**	.9	(.6,1.2)	.341
Age 18–25	**1.4**	**(1.0, 2.0)**	**.04**	1.5	(1.1, 2.1)	**.019**	.9	(.7, 1.1)	.264
Less than high school education	.8	(.5, 1.3)	.402	1.1	(.7, 1.7)	.612	.9	(.6, 1.3)	.545
Poverty	**1.5**	**(1.02, 2.3)**	**.042**	1.2	(.8, 1.8)	.376	1.2	(.8, 1.7)	.323
Injection drug use									
Heroin	Ref	Ref	Ref	Ref	Ref	**Ref**	Ref	Ref	**Ref**
Cocaine	.9	(.6, 1.7)	.958	1.3	(.8, 2.3)	.321	.7	(.5, 1.0)	.070
Heroin and cocaine	1.1	(.7, 1.8)	.632	1.5	(.9, 2.4)	.072	2.3	(1.6, 3.4)	**< .001**

Bold indicates significance at *p* < .05

*p < .05, **p < .01, ***p < .001

## Discussion

Findings from this study support hypotheses that obtaining syringes from SEPs and pharmacies would be associated with lower odds of syringe sharing and reusing syringes compared to obtaining syringes on the street. People who reported obtaining syringes at pharmacies and SEPs were less likely to engage in syringe sharing and reusing syringes compared to people who obtained syringes on the street. PWID who obtain syringes on the street may be more likely to reuse syringes and mitigate potential harm by sterilizing syringes with bleach prior to injection. Findings from this research are congruent with prior cohort studies suggesting that obtaining syringes from SEPs reduce syringe sharing behaviors [10, 16, 17, 38]. This study supports findings from a smaller number of studies focusing on the protective association between obtaining syringes at pharmacies and syringe sharing behaviors [8, 11],This study is the first study using nationally representative data of the U.S population to examine the protective associations between obtaining syringes at pharmacies and SEPs. This study underscores the need to scale up and expand the provision of sterile syringes in the United States, particularly in pharmacies which have traditionally been an underutilized resource in HIV prevention efforts [8]. The strong associations between cleaning and reusing syringes with bleach and engaging in syringe sharing further underscores the need to expand SEPs and pharmacy access to sterile syringes. PWID who obtain syringes on the street may be more likely to reuse syringes and mitigate potential harm by sterilizing syringes with bleach prior to injection. A potential explanation of this would be that PWID sterilize with bleach as a strategy of practicing harm reduction in contexts of poor access to sterile syringes.

These findings fortify existing evidence of the harm reduction utility of expanding SEP and pharmacy access for people who inject drugs. Although cleaning syringes with bleach is more effective than no cleaning at all, often bleach inadequately sterilizes syringes leaving significant risk of transmission of viral and bacterial infectious diseases. Obtaining a syringe at a pharmacy was significantly associated with reusing syringes. Future research is needed using nationally representative data that examines the impact of state-level policies, and clinic practices on access to sterile syringes among PWID. Syringe exchange programs are legal in 33 states-yet SEP often exist under the protection of city memoranda and other policies. The criminalization of injection drug paraphernalia in state and local jurisdictions and aggressive policing practices may discourage PWID from accessing syringes from SEPs and pharmacies [5, 9, 22, 33]. At the clinic level, prior research suggests that many pharmacists are reluctant to sell syringes to PWID and require education to reduce stigma and increase knowledge about the laws surrounding syringe provision [3, 18, 24, 28, 29].

### Limitations

There are several limitations worth noting. We used pooled cross-sectional data thus precluding any causal inference. The dependent variables only included two forms of syringe sharing and the questionnaire did not measure sharing involving equipment (i.e. cookers, cotton etc.). Often PWID dissolve heroin and other drugs into the same solution and draw injection fluid from the same container into different syringes. This form of sharing cookers, injection fluid and cotton introduce pathways to HIV infection not included in the data used for this study. Harm reduction practices (using bleach to clean last needle, where needles were purchased, etc.) were measured based on last injection rather than a numerical indicator within a specific time period.. One important consideration is that the subgroup of people who utilize SEPs may inject more frequently than the average person who injects drugs thus contributing to the lack of significant associations with obtaining syringes at an exchange program in the adjusted regression models [35].

Another limitation of this study is that it was not possible to know legal status of pharmacy sale of syringes and SEPs in the state of residence of participants. Future research must control for state-level confounds that include legal status of harm reduction programs promoting syringe access. We did not adjust for SEP/pharmacy access policies across geography. States in the U.S have wide-sweeping variation in state-level policies governing SEP and pharmacy access which could exert great influence over syringe sharing behaviors and harm reduction practices. Finally, this study did not examine patterns in syringe sharing behaviors by social status variables which would provide some richer information. Future research is critically needed that captures how the policy environment shapes harm reduction behaviors at the individual level. A fruitful avenue of future research is to further examine differences in the relationships between sources of syringes and syringe sharing across types of drugs used. There is a rapidly emerging body of literature suggesting burgeoning rates HIV among people who inject both stimulants and heroin. Future research must examine differences by syringe sharing behaviors and social status variables including education, income as well as the interaction of social status variables and race/ethnicity.

### Implications for future research

Several fruitful implications for future research arise out of findings from this study. Future research must adjust for frequency of injecting to differentiate if insignificant results were not spuriously due to differences in frequency of injecting rather than direct relationships between SEP access and sharing syringes. Greater research is needed that examines racial and ethnic differences, as well as differences by social status such as education and income in rates of access to SEPs, pharmacies and obtaining syringes on the street. PWID who are Black and Hispanic may have less access to SEP and pharmacy distribution of syringes resulting in greater rates of syringe sharing. Findings from this study support the need for future research that measures more systemic and structural factors that may affect access to SEPs and pharmacies such as stigma fueled by racism and criminalization [12]. Prior research suggests that PWID from minority populations experience intersecting structural forms of racism and stigma including negative treatment from providers, harassment from law enforcement officers, and greater public scrutiny resulting in exclusion from access to syringe exchange programs (SEP) and pharmacies [4, 7, 12, 23]. Prior research suggests that pharmacies located in neighborhoods that are impoverished with lower rates of insurance coverage are less likely to be supportive of harm reduction strategies. Future empirical inquiry must continue this research by examining the interaction between structural forms of racism and discrimination, violence by the police and access to SEPs and pharmacies. Future research is critically needed that investigates why people choose particular harm reduction strategies with respect to reusing/lending syringes vs. getting new syringes that takes into account structural constraints such as policies, gaps in regional coverage by programs and other factors.

## Conclusion

Limitations aside, findings from this study support expanding SEPs and syringes provided by pharmacies to reduce syringe sharing. Amidst a growing number of PWID in the United States additional research is needed to identify new and innovative methods of syringe distribution that are associated with decreased engagement in sharing and reusing syringes to increase the uptake of syringe access for the approximately 1 million people who inject drugs in the US.

## Data Availability

Data for this study consisted of publicly archived available data from 2002–2019. Data can be obtained from: https://www.samhsa.gov/data/data-we-collect/nsduh-national-survey-drug-use-and-health. The datasets used and/or analyzed during the current study are also available from the corresponding author on reasonable request.

## Abbreviations

PWID: People who inject drugs
HCV: Hepatitis C
HIV: Human Immunodeficiency Virus
CDC: Centers for Disease Control
SEP: Syringe exchange program
NSDUH: National Survey of Drug Use and Health
SAMHSA: Substance Abuse Mental Health Services Administration
(a)OR: (Adjusted) odds ratio
CI: Confidence interval
